# Effects of Acupressure on Maternal and Neonatal Obstetric Outcomes during Labor: Study Protocol

**DOI:** 10.3390/healthcare11142111

**Published:** 2023-07-24

**Authors:** Miguel Ángel Carmona-Rodríguez, Salvador Martínez-Flores, Rubén Morilla-Romero-de-la-Osa, Manuel Luque-Oliveros

**Affiliations:** 1 Midwifery Training Unit, Department of Materno-Fetal Medicine, Genetics and Reproduction, Hospital Universitario Virgen del Rocío, Avda. Manuel Siurot s/n, 41013 Seville, Spain; miguel.carmona.rodriguez.sspa@juntadeandalucia.es; 2 Hospital Materno-Infantil Quirón Sagrado Corazón, 41013 Sevilla, Spain; 3 Departamento de Enfermería, Facultad de Enfermería, Fisioterapia y Podología, Universidad de Sevilla, 41009 Sevilla, Spain; mluque5@us.es; 4 Servicio de Cirugía Cardiovascular y Área del Corazón-Cirugía Torácica, Hospital Universitario Virgen Macarena (HUVM), 41009 Sevilla, Spain; 5 Instituto de Biomedicina de Sevilla (Hospital Universitario Virgen del Rocío/CSIC/Universidad de Sevilla), 41013 Seville, Spain; 6 Centro de Investigación Biomédica en Red de Epidemiología y Salud Pública (CIBERESP), Instituto de Salud Carlos III, 28029 Madrid, Spain

**Keywords:** labor, pain, acupressure, midwifery

## Abstract

Background. A Cochrane review found that there is insufficient evidence to determine the effectiveness of acupressure for pain relief. One of the problems detected is the methodological variability reported. Objective. To assess the impact of the application of acupressure on obstetric and neonatal outcomes of labor, pain experience, and mother’s satisfaction with the experience. Method. Design of a protocol to carry out a two-arm multicenter single-blinded randomized controlled trial. Intervention (pressure on LI4 of the left hand, B6 of the left leg, GB21 of the left shoulder, and then the same sequence on the right side) and placebo (application of the technique on points not identified by acupuncture as key points) will be performed by a single researcher. Results. The recruitment began in April 2021 and, to date, there has been the participation of 40 women, divided into 17 included in the experimental group and 23 in the control. Communication of future results will be made in accordance with the CONSORT checklist. Conclusions. The designed protocol could methodologically improve some aspects of previous studies while maintaining adequate statistical power. The effectiveness of acupressure for one or more outcomes proposed (time and pain in labor) could support the inclusion of a new therapeutic tool in the clinical practice of midwives that would allow them to assist pregnant women, improving their experience both physically and psycho-emotionally

## 1. Introduction

Childbirth, being a natural phenomenon, encompasses various dimensions, making it a comprehensive and subjective experience in which physiological, psychological, social, and cultural factors come into play [1]. The perception of pain during the process is a key aspect, and it is worth noting that factors such as fear of pain, concerns about undergoing an episiotomy, and the fear of losing control over the situation have been identified as significant contributors to tokophobia, which is a psychological condition characterized by an intense fear of childbirth [2].

It is noteworthy that women exhibit a keen interest in exploring complementary forms of care that empower them to maintain a sense of control throughout the labor process [3]. Midwives also want offer alternatives and, consequently, non-pharmacological techniques for pain management during childbirth, as well as methods for labor induction or stimulation, have emerged as potential alternatives or supportive measures in the context of medicalized childbirth [4]. These approaches hold promise in providing women with additional options and support during childbirth, fostering a holistic approach to the birthing experience.

Different techniques developed in traditional Chinese medicine are used as alternative and/or complementary therapies in order to reduce the medicalization of healthcare. Some of these most-used techniques are acupuncture, acupressure, moxibustion (moxa), and transcutaneous electric nerve stimulation (TENS) on acupoints, and all have been applied to pregnant women for this purpose [5]. Within the realm of traditional Chinese medicine (TCM), qi represents the vital energy that traverses the body’s 14 pathways, commonly known as meridians. By employing techniques such as acupuncture and acupressure, it becomes possible to stimulate this energy and reinstate equilibrium and well-being. These techniques arose from the “gate theory”, in which it is believed that the stimulation of these points can stimulate the fibers that transmit impulses to the spinal cord. Specifically, acupressure consists of stimulating acupuncture points through the hands and/or fingers, as well as utensils such as awls and even ice [4,6].

The recent literature has provided affirmations regarding the potential benefits of applying pressure on acupuncture points in improving obstetric and neonatal outcomes. While there is a growing consensus on the effectiveness of this technique in reducing perceived pain during pregnancy, further investigations are still warranted to explore its impact on labor duration, maternal satisfaction, and associated neonatal outcomes. These types of outcomes are less explored and when, they are evaluated, they can be studied with different methods or partial and inconclusive results can be obtained [7,8,9,10].

Building upon this foundation, Haj and Xiao-Nong emphasized the need for standardized interventions to establish clear guidelines for the utilization of this technique and to demonstrate its utility [9]. Notably, a comprehensive Cochrane review conducted by Smith et al. in 2020 reported a lack of studies that conclusively confirm the positive impact of acupressure on pain relief due to limited available evidence. The review also identified challenges associated with variability in the selection of acupuncture points, diverse techniques employed, inadequate information on applied pressure and duration, as well as differences among therapists, which can introduce further variations in practice [10]. It is evident that further research and standardization efforts are essential to shed more light on the potential benefits and enhance the utilization of acupressure as a complementary approach in obstetric care.

Some of the most-used points in the literature were:

- Jianjing (GB21): Located in the middle of the imaginary line that joins the spinous process of the seventh cervical vertebra (C7) and the upper part of the shoulder joint (lateral end of the acromion). It is a point with sensation of heat or numbness. It is a point that reacts to pressure, and can generate an unpleasant sensation. This point is useful in the stage of fetal dilation and descent, and can stimulate uterine contractions [11,12,13].
- Hegu (LI4): Located between the first and second metacarpals (thumb and index bones). It is at the highest point seen when the thumb rests on the index finger. It acts as a stimulus for contractions if they are irregular, making them more efficient [14,15,16,17,18].
- Sanyinjiao (B6): It is located four fingers of the woman above the prominence of the medial malleolus (inside of the ankle). It is a pressure-sensitive area. This point helps the cervix to dilate more easily. Pressing can be stopped once labor has effective and regular contractions [1,11,12,15,16,19,20].
- Yongquan (R1): It is located in the depression found in the upper third of the sole of the foot (easily located when the foot is placed in plantar flexion, that is, flexing the fingers towards the sole of the foot). It is essential to apply forceful pressure, squeezing inwards and upwards, towards the first toe. It has a relaxing effect [21].
- Hand Points: These are located along the creases of the hands where the fingers join the palm. Helps release endorphins, which are natural pain relievers. The woman holds a small comb in the palm of her hand so that the teeth press on these points. She can squeeze the comb during contractions by pressing to get the desired effects [21].

In short, labor and the pain associated with this phase is something that worries pregnant women, manifested by the high demand for pharmacological methods for pain relief, so identifying non-pharmacological interventions that reduce or minimize this will allow a significant improvement in women’s satisfaction with the vital experience of childbirth. The midwife constitutes the cornerstone to achieve this purpose [22].

## 2. Objectives and Hypothesis

The general objective is to assess the impact of the application of acupressure on obstetric and neonatal outcomes of labor. To achieve the general objective, four specific objectives have been established, each based on the verification or refutation of a specific hypothesis.

Objective 1: To analyze the possible statistical association between the use of acupressure and the times of cervical dilation and fetal delivery.

- Null hypothesis: The dilation time and the expulsive time in the women who receive the intervention is the same as those of the women in the control group.
- Alternative hypothesis: The time of dilation and the time of expulsion in the women who receive the intervention is not the same as those in the women in the control group.

Objective 2: To compare the pain perceived by pregnant women in relation to the use of the acupressure technique.

- Null hypothesis: The pain experienced by women receiving the intervention is the same as that experienced by women in the control group.
- Alternative hypothesis: The pain experienced by women receiving the intervention is not the same as that experienced by women in the control group.

Objective 3: To study the relationship between the use of acupressure and maternal satisfaction regarding the childbirth experience.

- Null hypothesis: The satisfaction experienced about the birth process in women receiving the intervention is the same as that in women in the control group.
- Alternative hypothesis: The satisfaction experienced about the birth process in women who receive the intervention is not the same as that in women in the control group.

Objective 4: Assess the safety of the intervention through fetal and neonatal outcomes.

- Null hypothesis: The fetal and neonatal results of the pregnant women belonging to the intervention group are the same as those in the pregnant women of the control group.
- Alternative hypothesis: The fetal and neonatal results of the pregnant women who belong to the intervention group are not the same as those in the pregnant women of the control group.

## 3. Materials and Methods

### 3.1. Setting

The primary investigator leading this study is an experienced and dedicated midwife who actively practices in a private hospital, along with regular rotations through multiple public hospitals. This diverse practice setting offers a valuable opportunity to include pregnant women from at least three distinct health centers, encompassing a wide range of profiles and characteristics among the participants.

### 3.2. Design

This study employs a robust multicenter randomized controlled single-blind experimental design. The intervention group will receive acupressure applied to specific points that will be thoroughly explained later in the study. As for the control group, acupressure will be administered to points that, according to traditional Chinese medicine’s energy meridians, are deemed to lack the desired therapeutic effect. Due to the fact that the principal investigator is directly responsible for both administering the intervention and the placebo, the study cannot be designed as a double-blind trial. To ensure transparency and impartiality, the collected data will be provided anonymously to a secondary investigator who will conduct the statistical analysis without any indication of which group represents the control or intervention.

### 3.3. Sampling and Sample Size

The sampling process will be conducted using an intentional or convenience sampling approach, wherein all pregnant women who meet the inclusion criteria and are under the care of the project’s principal investigator will be included until the predetermined sample size is reached. This meticulous approach aims to encompass a diverse range of participants, thereby enhancing the generalizability of the study findings.

The calculation was based on the results obtained in a pilot study (unpublished data) with 17 and 23 women in the intervention and control groups, respectively, using the Gpower 3.1.9.4 software. Different calculations were carried out based on the means and standard deviations obtained in the times of the active, passive, and expulsive phases, as well as on the VAS scale (Likert 1–10 points) used to measure pain. From the different calculations carried out, the one that determined the largest sample size (difference of means in time of expulsive phase) was selected with the intention of having sufficient statistical power for all the analyses proposed.

The means and standard deviation obtained for the expulsive phase time (second stage of parturition, which starts at full dilation and continues until birth) in a pilot study were 46 ± 39 min for the intervention group and 65 ± 47 min for the control group. The number of women per group was calculated to determine a difference in the means of independent groups with alpha = 0.05 (type I error), power (1-beta) = 0.8, and allocation n1/n2 ratio = 1, obtaining a total of 166 women (83 in each group) [23].

Recruitment. The selection of participants for this study will adhere to specific criteria outlined as follows:

Inclusion criteria: The study will include pregnant women who meet the following requirements:

1. Women with induced, accelerated, or spontaneous labor, both primiparous and multiparous, are included.
2. Women who are 18 years of age or older and in the term of their pregnancy (37–42 weeks).
3. Single gestation (not multiple pregnancies).
4. Absence of associated pathologies or medical conditions.
5. Proficient communication skills in the Spanish language.

Exclusion criteria: The study will exclude pregnant women who meet any of the following criteria:

1. Women with a fetus in a breech or transverse presentation.
2. Women who have previously undergone caesarean sections.
3. Women with existing skin lesions or wounds.
4. Women with cognitive deficits or impairments.
5. Women who have consumed toxic substances or medications during their pregnancy.
6. Women with diagnoses of placenta previa (previous insertion placenta), fetal pathologies such as Delayed Intrauterine Growth (DIC) or Small for Gestational Age (SGA), or instances of oligo- or polyhydramnios.

These well-defined criteria will ensure that the study focuses on a specific group of pregnant women, enabling researchers to examine the intended effects of the intervention more accurately. 

Allocation. Assignment to the intervention group will be made by simple randomization of the sample. To do this, a random sequence of numbers will be generated using R software (R 4.1.3, March 2022), which is open source and specifically designed for statistical analysis, to assign women to intervention/control groups. For this purpose, the function will be used: sample (1:166, 83, replace = FALSE). The women who are attended in the order of arrival will be included in the intervention group if their order coincides with any of the random numbers generated.

Intervention. Intervention will be implemented by a single researcher and it will be based on pressure on the points detailed below:

It will start by exerting pressure on:

- LI4 of the left hand, SP6 of the left leg, GB21 of the left shoulder, and then the same sequence on the right side. This is intended to generate regular and effective contractions and promote the descent of the fetal head.
- Yongquan (R1): For pain relief and relaxation of women, exerting constant pressure for 1 min, as many times as necessary to promote pain control.
- Hand points: Also for pain relief, autonomously by the woman, whenever she needs it.

The location of the trigger points can be visualized in Appendix A.

Constant pressure will be generated for 3 min, alternating points, and repeating the technique every 4 h, after the scans. Digital pressure will be applied until half of the nail turns white (equivalent to 3 to 5 kg of pressure) [23]. The Hegu point (LI4) will also serve as an analgesic point.

Based on the research carried out by Dávila Payano and Lazo del Carpio (2016), as for the main researcher, it is essential to maintain a deep and relaxed breath, and to apply deep and lasting pressure, bearing all the weight on the pressure point during the exhalation. Your position will be static, standing, with legs slightly apart, exerting force with your body weight. The manipulation must be constant, energetic, uniform, and delicate, using fingers or knuckles [21].

Control. The control group will receive the application of the technique on points not identified by acupuncture as key points (placebo), but with the same characteristics described above (time, frequency, pressure). Different points will be used as placebo points and will be determined using some of the potentially therapeutic points as references; some of these points will be one F-Cun away from the original points with therapeutic effect. The Cun measurement, derived from acupuncture anatomy, represents the width of the thumb at the joint level and serves as an important anatomical unit in traditional acupuncture practices. In traditional Chinese medicine and acupuncture, the concept of Cun plays a vital role in determining precise anatomical locations for treatment. It is a unit of measurement utilized to establish the distances between acupuncture points on the human body. The Cun measurement is unique to each individual and is derived from the width of the thumb at the joint level. By using the Cun measurement as a standardized reference, acupuncturists can accurately locate specific points for therapeutic intervention. In this study, the F-Cun distance will be employed as a reference point for the placement of placebo points, ensuring consistency and precision in the experimental design [23]. The control points placed an F-Cun away from real acupoints will be GB21 (on the shoulder) and another one will be placed between the knuckles of the index and middle fingers. Additionally, the last one will be 2 F-Cun higher than the SP6 placement (see exact placement in pictures at the end of this paper).

The purpose behind administering pressure to different points using fingers in the control group is to eliminate any potential placebo effect associated with “manipulation using a therapeutic instrument”. It is important to note that none of the points pressed in either the intervention group or the control group possess an anatomical location that can induce significant physiological changes when pressure is applied, such as pressing the carotid artery. This precautionary measure ensures the safety of the study participants, minimizing any adverse effects resulting from the applied pressure on the women. Thus, the inclusion of these additional control points not only serves to rule out placebo effects, but also guarantees the well-being and welfare of the participants by preventing any unintended consequences arising from the pressure applied during the study.

Outcomes. Variables will be measured in order to assess the efficacy and safety of the intervention:

- Effectiveness of the use of acupressure on labor times in minutes (time of cervical dilatation and time of fetal expulsion according to the recommendations of the World Health Organization).
- Effectiveness of the use of acupressure on women’s perceived pain by recording values on the VAS scale and the need for pharmacological analgesic methods. A Visual Analogue Scale (VAS) is a pain rating scale that was first used in 1921 by Hayes and Patterson. The pain VAS is a one-dimensional measure of pain intensity that is used to record the progression of patients’ pain or to compare the severity of pain between patients with similar conditions. The patient must rate their perceived pain on a scale of 0 (no pain) to 10 (the most severe pain imaginable) [1,4,13,14,15,17,19,24,25,26].
- Impact of the use of acupressure on maternal satisfaction expressed about the childbirth experience (using the validated satisfaction scale Mackey Satisfaction Childbirth Rating Scale—MSCRS) [8].
- Impact of the use of acupressure on neonatal outcomes (Apgar test at 1/5/10 min and arterial blood gases at birth).

Independent variables.

- Sociodemographic: Age (years), country of birth, weight (kg), height (cm), marital status (single, domestic partner, married, divorced, widowed), studies (primary, secondary, superior, postgraduate), employment (employed, unemployed), dysmenorrhea: yes/no (verbalization by the woman of having painful menstruations), fear of childbirth (yes/no), reasons for fear of childbirth (verbalization by the woman), hospital (public/private).
- Clinics: date of last period (FUR) and probable date of delivery (FPP), gestational age (weeks of gestation), obstetric formula (pregnancies, abortions, childbirth), estimated fetal weight (kg), fetal weight at birth (kg), type of delivery termination (eutocic, instrumented, caesarean section), special circumstances associated with childbirth (maternal fever, meconium, cord prolapse, antepartum hemorrhage, and uterine rupture), factors associated with the newborn (abnormal registration and altered arterial pH, considering normal values—arterial pH at birth: 7.25–7.45, blood oxygen (PaO2): 60–80 mmHg, blood carbon dioxide (PaCO2): 35–45 mmHg, bicarbonate (HCO3-): 24–26 mEq/L, base excess: ±3.0).
- Variables that can affect the effectiveness of the intervention: hospital shift the night before (yes/no), start time of application of the technique (0–24 h), level of saturation of the system (number of women in the service attended by the researcher).

Data analysis. The data obtained will be analyzed with IBM SPSS Statistics v23 software; this will allow for the descriptive analysis of qualitative variables through absolute frequencies and percentages, and of quantitative variables through measures of central tendency, dispersion, and position. On the other hand, a statistical inference analysis will be carried out to evaluate the possible differences between the groups; these will be treated as unpaired samples because there are different women who constituted each group.

The statistical association between qualitative variables will be analyzed using Pearson’s chi-square test or Fisher’s exact test (in 2 × 2 tables when in Pearson’s Chi square, the expected frequencies are less than 5 in more than 20% of the cells). Odd ratio will be calculated as size effect measure for this analysis.

For the comparison of means between dichotomous variables, the Student t-test and ANOVA test will be performed in the case of dichotomous or polytomous variables, respectively, as long as the parametric criteria are met (normality evaluated using the Kolmogorov–Smirnov test and homoscedasticity evaluated with the Levene test). If the parametric criteria are not met, non-parametric tests will be performed (Wilcoxon test and Kruskal–Wallis test, respectively). For the Student t-test analysis, Cohen’s d will be used as a measure of the effect size. 

For regression analysis, the Pearson correlation coefficient will be used. In the case of non-normal distributions, Spearman correlation test will be used. Statistical significance at 5% will be established for all tests.

Ethical aspects. The present study preserves the well-being of the subject above the interests of science or society and the ethical considerations that support it, as part of the compliance with current international and national regulations for health research studies with human beings, as well as that referring to the protection of personal data and clinical information derived from it. Under these premises, this protocol has been approved by the Regional Ethics Committee (committee code 4928, project code: AcuP, approval date 24 February 2021, record number 319).

Data collection notebooks will be handled in which only identification codes will appear that will correspond to the personal data in other guarded records. Similarly, electronic database-type records will be designed disaggregated to avoid identification of patients. An Informed Consent document has been prepared. The intervention does not require invasive tests or manipulation of biological samples beyond those implemented in routine clinical practice.

## 4. Results

The recruitment and inclusion of women in the study began in April 2021 and, to date, there has been the participation of 40 women, divided into 17 included in the experimental group and 23 in the control. Among them, 95% of the women were treated in a private hospital. They are Spanish women (so far only one woman of Chinese, one of Moroccan, and one of Portuguese nationality have been treated), around 33 years of age, married, with higher education, gestation at term and without complications, with an estimated fetal weight around 3330 kg and with fear of childbirth whose stated reasons were: pain (35%), ignorance (20%), epidural, completion of labor, and loss of control, with 2.5% each. 

To communicate the results, the CONSORT checklist [27] and its flowchart will be used, in order to use a validated instrument whose objective is transparent and free (or minimized) communication of methodological biases.

## 5. Discussion

The primary objective of this study’s protocol is to introduce a novel acupressure intervention, incorporating points that have been extensively studied in the scientific literature and have demonstrated their effectiveness. However, certain points located on the back were deemed impractical due to the supine position in which women typically find themselves during childbirth. Consequently, these points were excluded from the intervention. Conversely, we have identified points from Dávila’s doctoral thesis that have not been previously documented [21]. The strategic positioning of these points enables a more practical and feasible intervention approach. 

Based on the findings, it is anticipated that the meticulously chosen set of points, previously unexplored, will provide a prompt and effective intervention. We expect this intervention to yield improved perinatal outcomes while minimizing discomfort for the mother. By utilizing these previously unexplored points, we aim to optimize the intervention’s efficacy and enhance the overall childbirth experience for women. Therefore, the midwife should position the woman in the standard childbirth posture and can proceed with the intervention, as easy access to the proposed points is ensured in this position. This is crucial from an ergonomic perspective, benefiting both the pregnant woman and the midwife.

The outcomes derived from the implementation of this protocol possess the potential to generate significant and applicable insights for clinical practice, thereby enhancing the overall childbirth experience by reducing pain perception and duration. Consequently, such improvements would directly contribute to heightened satisfaction among women undergoing this crucial life event. Furthermore, if the safety of the intervention for both mothers and infants is demonstrated, its incorporation into the healthcare system of our country would carry substantial weight as a valuable tool for future implementation.

Nevertheless, it is important to acknowledge certain limitations that may arise in relation to the sample size. The current study is based on a small, unpublished pilot study that was organized ad hoc to collect data for the development of this protocol. However, we firmly believe that our meticulous approach will yield more robust scientific evidence compared with previous studies that lacked explicit mention of sample size calculations [14,28] or studies that briefly referenced sample size calculations without providing specific details [1,17,26,29,30,31].

It is noteworthy to highlight that our sample size aligns closely with those observed in other studies published in high-impact nursing journals, reinforcing the methodological robustness of our research [23]. Lastly, we consider the design of a multicenter study with a single researcher responsible for administering the intervention to be a methodological triumph. This approach ensures a greater diversity within the sample population, mitigating potential variations in clinical practices and, to some extent, bolstering external validity.

## 6. Conclusions

If the results of this study confirm the effectiveness of acupressure in achieving the proposed outcomes outlined in the research hypotheses, it would provide substantial support for the integration of this therapeutic modality into the clinical practice of midwives. The inclusion of acupressure as a viable tool would empower midwives to offer comprehensive care to pregnant women, enhancing their overall experience not only on a physical level, but also in terms of their psycho-emotional well-being.

By incorporating acupressure as a part of their practice, midwives would be equipped with an additional evidence-based intervention to address various aspects of pregnancy, labor, and childbirth. This holistic approach to care has the potential to optimize maternal comfort, promote a sense of control and empowerment, and foster positive emotional experiences during this transformative journey.

Furthermore, the integration of acupressure into midwifery practice aligns with the growing recognition of the importance of complementary and alternative therapies in maternity care. It would provide midwives with a valuable tool that complements their existing knowledge and skills, enhancing their ability to provide personalized and holistic care to pregnant women.

Ultimately, the successful implementation of acupressure as a therapeutic intervention within midwifery practice has the potential to positively impact not only the physical well-being of pregnant women, but also their overall satisfaction and emotional connection to the childbirth process. By recognizing and embracing the potential benefits of acupressure, midwives can contribute to a more positive and empowering childbirth experience for women, promoting their overall health and well-being.

## Figures and Tables

**Figure A1:**
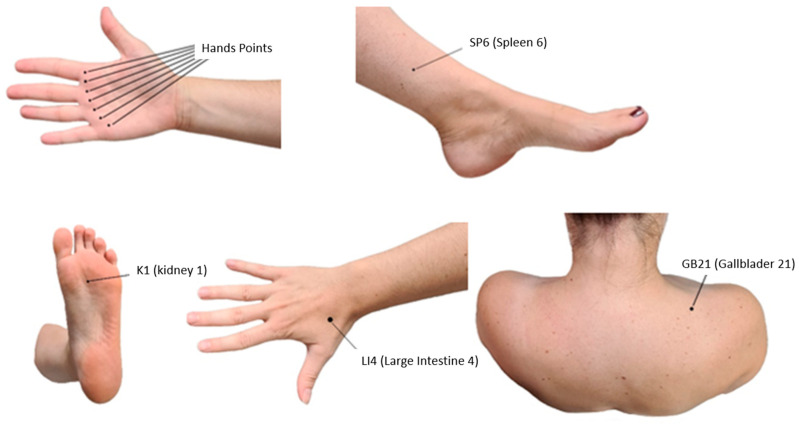
This appendix shows the location of the trigger points selected to create this new protocol.

## Data Availability

Not applicable.
